# Prevalence and Risk Factors of *Opisthorchis viverrini* Infection in Sakon Nakhon Province, Thailand

**DOI:** 10.3390/tropicalmed7100313

**Published:** 2022-10-18

**Authors:** Pariyakorn Perakanya, Ratchadaporn Ungcharoen, Sutthiporn Worrabannakorn, Passakorn Ongarj, Atchara Artchayasawat, Thidarut Boonmars, Parichart Boueroy

**Affiliations:** 1 Department of Community Health, Faculty of Public Health, Chalermphrakiat Sakon Nakhon Campus, Kasetsart University, Sakon Nakhon 47000, Thailand; 2 Cholangiocarcinoma Research Center, Sakon Nakhon Hospital, Sakon Nakhon 47000, Thailand; 3 Department of Parasitology, Faculty of Medicine, Khon Kaen University, Khon Kaen 40002, Thailand

**Keywords:** *Opisthorchis viverrini*, Thailand, risk factors, prevalence

## Abstract

Opisthorchiasis is a parasitic infection caused by the liver fluke *Opisthorchis viverrini*. This parasite is widely distributed and well documented in Thailand, Lao PDR, Southern Vietnam, Cambodia, and Myanmar. However, its prevalence is a major problem in these countries. Thus, the aim of this study was to determine the prevalence and risk factors of *O. viverrini* infection from 2017 to 2020 in Sakon Nakhon province, Thailand. Questionnaires were used to interview 320 participants (160 cases and 160 controls) in a random selection of 18 districts across Sakon Nakhon province. Univariate logistic regression was used to identify the factors associated with *O. viverrini* infection. The overall prevalence levels of *O. viverrini* infection in Sakon Nakhon province for 2018, 2019, and 2020 were 3.60%, 5.21%, and 7.01%, respectively. Raw fish consumption was a positive risk factor for its infection in endemic areas. Factors associated with *O. viverrini* infection were the habit of consuming unsafely prepared fish (OR = 6.33, 95%CI = 0.32–0.59), the medical history of *O. viverrini* examination (OR = 8.93, 95%CI = 5.15–15.47), a history of *O. viverrini* infection (OR = 3.64, 95%CI = 1.17–1.44), and a history of taking praziquantel (OR = 3.64, 95%CI = 1.17–1.44). These results identified gaps in the epidemiological knowledge of *O. viverrini* in this region that need addressing to identify and develop innovative methods for prevention, control, and support efforts to permanently overcome *O. viverrini* infection in endemic regions.

## 1. Introduction

Opisthorchiasis is a fish-transmitted trematodiasis, caused by infection with members of the trematode family Opisthorchiidae, including *Opisthorchis viverrini*, *Opisthorchis felineus*, and *Clonorchis sinensis* [1]. This disease is a major public health problem in five countries in the Greater Mekong Subregion: Thailand, Laos, Myanmar, Cambodia, and Vietnam [2]. People living near the Mekong river created a higher risk of *O. viverrini* infection than mountain dwellers [3,4]. Opisthorchiasis is difficult to control because there is a complex life cycle which includes several hosts, environments, and a complicated disease transmission process [5]. In Thailand, at least 6 million people are infected with the liver fluke *O. viverrini* [6]. The geography of this region is highly variability among provinces, with *O. viverrini* infection rates reported to vary between 4.6% and 60% [7]. Chronic infection of *O. viverrini* is associated with hepatobiliary diseases and cholangiocarcinoma (CCA), which is a primary liver cancer in this region [8]. The medical care and loss of wages resulting from *O. viverrini* infection in Thailand costs approximately USD $120 million annually [9]. 

Sakon Nakhon province is a province in the northeast region of Thailand with historically very high rates of *O. viverrini* infection and, specifically, the prevalence rate of cholangiocarcinoma (CCA) has been higher than in any other province in this region [8]. The tradition of eating raw, fermented, pickled, and undercooked cyprinid fish are a significant risk factor and the primary source of liver fluke infection [8]. Every year over 1000 new cases of CCA are identified in Sakon Nakhon hospital; this incidence has not declined over the decades despite the major risk factors of *O. viverrini* infection being known [10]. Another study has reported that the incidence of CCA in the four major regions of Thailand (Sakhon Nakhon, Prae, Roi-Et, and Nongbua Lampoo) has strong correlation with the prevalence of *O. viverrini* infection [11]. People with high *O. viverrini* infection intensity (>6000 egg/gram feces), were 14.1 times (odds ratio) more likely to develop CCA than people who were not infected [12,13]. The humans who get the *O. viverrini* infection have been develop to CCA around 10%, creating a serious health emergency throughout the region [3]. The effective treatment for *O. viverrini* infection is praziquantel with a single dose of 40 mg/kg. The cure rate of praziquantel is as high as 95.5%. Some individuals ignore this information because there was no need to know about the treatment if they were never infected [14]. The *O. viverrini* infection can produce hepatic bile ducts and portal connective tissue inflammation resulting in CCA development [4,15]. The five-year survival rate of intrahepatic, distal extrahepatic, and hilar CCA patients that receive surgical intervention are 22–44%, 27–37%, and 11–41%, respectively [16]. The survival rate of CCA patients for ages 30–40, 41–45, 51–60, and 61–98 years was 22.3% (95% CI, 16.8–29.5), 14.3% (95% CI,12.0–17.0), 8.6% (95% CI, 7.8–10.0), and 7.2% (95% CI, 6.4–8.0) [17]. The results demonstrated that the survival rates of CCA patients were poor [17]. Although there are well-documented risk factors for *O. viverrini* infection and CCA in the northeast region, this information needs to be updated and confirmed for Sakon Nakhon, Thailand.

The Thai Ministry of Public Health has published three strategic guidelines for the prevention and control of *O. viverrini* infection in Thailand: (1) stool examination; (2) health education; and (3) the treatment of *O. viverrini* infection cases with praziquantel [18,19]. In 2017, the Thai Ministry of Public Health announced plans to eradicate *O. viverrini* infection and CCA. This program was a collaboration with global organizations such as the Cholangiocarcinoma Foundation USA, AMMF UK (The Cholangiocarcinoma Charity), the Thailand Cholangiocarcinoma Foundation, and the Cholangiocarcinoma Screening And Care Program (CASCAP) of Khon Kaen University, Thailand [20]. The project was developed the cloud real-time database called “Isan-cohort” to screen and collect data for northeastern and eastern Thailand [20]. 

The Thai government has run a campaign to restrict this disease since the 1980s; however, the prevalence of *O. viverrini* infection has remained high and ongoing [7,21,22]. The obstacle for effective and sustainable control of *O. viverrini* infection in northeast Thailand has been identified, with a lack of knowledge of the disease being a major problem in the effective control of this disease [23]. Therefore, active and effective surveillance in rural communities and the characterization of population risk factors associated with *O. viverrini* infection are needed to reduce the risks of this infection and the incidence of CCA. Thus, the purpose of this study was to investigate the population risk factors and to identify the factors associated with *O. viverrini* infection among the population of Sakon Nakhon Province, Thailand.

## 2. Materials and Methods

### 2.1. Study Design

We conducted the retrospective cohort study from 1st October 2017 to 30th October 2020 to analyze the prevalence and risk factors of *O. viverrini* infection in Sakon Nakhon province, Thailand. The Research Ethics Committee approved this study by the Kasetsart University Chalermphrakiat Sakon Nakhon Campus (Kucsc.HE-64-009), the Sakon Nakhon Hospital (033/2564) and the Sakon Nakhon Provincial Public Health Office (018/2564).

### 2.2. Data Collection

We reviewed the clinical records of residents from 18 districts in Sakon Nakhon province that had attended the Cholangiocarcinoma Screening and Care Program (CASCAP), Thailand. The CASCAP is a project that includes screening patient cohorts by inclusion criteria for enrollment, and these are as follows: all residents of northeastern Thailand ≥40 years of age who have any of the following risk factors: infection with liver flukes, treated for liver fluke infection, or consumed raw freshwater fish. The case was based on examining stools for the presence of *O. viverrini* eggs using the Modified Kato Katz Thick Smear Technique. The demographic data and the questionnaire of probable risk factors were collected retrospectively. The sample size was calculated online based on EPITOOLS (https://epitools.ausvet.com.au/, accessed on 12 July 2022). Based on the expected proportion in controls = 0.5, odds ratio = 2.04 [24], confidence level = 0.95, and power = 0.80, the sample size per group = 122. Therefore, the desired sample size was 122 cases and 122 controls. For the current study, there were 160 cases and 160 controls, selected using simple random sampling with a random number table. In each year, 40 people were sampled who were infected with *O. viverrini*.

### 2.3. Statistical Analysis

The generated data set was exported to STATA program (version 17) for processing and analysis. The frequency and percentage statistics were used to summarize the dataset. Univariate logistic regression was used to develop a model to predict the occurrence of *O. viverrini*. This model was also used to identify the risk factors of *O. viverrini*. The test level of significance was 5%.

## 3. Results

**Figure 1:**
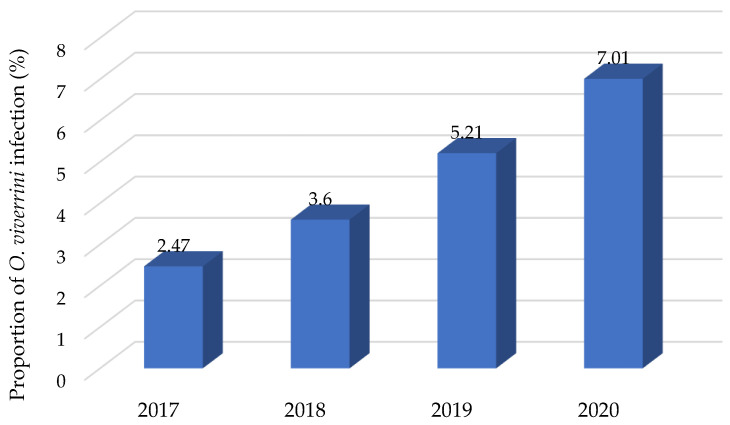
Prevalence of *O. viverrini* infection in Sakon Nakhon province, northeastern Thailand during 2017, 2018, 2019, and 2020.

The overall rates of *O. viverrini* infection during 2017, 2018, 2019, and 2020 were 2.47%, 3.60%, 5.21%, and 7.01%, respectively (Figure 1). The number of *O. viverrini* infections increased every year during the studied period. In total, 320 participants were included in this study. We reviewed 160 cases of *O. viverrini* infection, which included slightly more women (58.75%) than men (41.25%). Among the 160 studied cases, there were 27 CCA cases. The period for CCA identification in these cases was in the range 12–81 months after *O. viverrini* infection.

The baseline characteristics of the 320 participants are summarized in Table 1. Just over half (53.10%) of the total number of participants in the case and controls groups were female. Most participants were aged >55 (64.70%), married (92.20%), had graduated from primary school (90.90%), and were employed in agriculture (91.90%), while 58.80% reported that their family income was THB 5001–10,000 /month. Table 1 shows the univariate analysis results for the general factors contributing to the risk of infection with *O. viverrine* Age (OR = 1.50, 95%CI = 0.86–1.18), education level (OR = 1.28, 95%CI = 0.71–1.19), occupation (OR = 2.11, 95%CI = 0.61–1.11), and family income (OR = 1.16, 95%CI = 1.33–2.50) were not found to be risk factors, while gender (OR = 1.23, 95%CI = 0.84–1.15) and status (OR = 8.27, 95%CI = 0.55–1.05) were significant factors regarding *O. viverrini* infection in Sakon Nakhon province, northeastern Thailand.

The behavior factors associated with *O. viverrini* infection were the habit of eating raw fish several times, a history of *O. viverrini* examination and *O. viverrini* infection, and a history of taking praziquantel (Table 2). The associated factors of *O. viverrini* infection in Sakon Nakhon province, northeastern Thailand were the habit of eating raw fish (OR = 6.33, 95%CI = 0.32–0.59), a history of *O. viverrini* examination (OR = 8.93, 95%CI = 5.15–15.47), a history of *O. viverrini* infection (OR = 3.64, 95%CI = 1.17–1.44), and a history of taking praziquantel (OR = 3.64, (95%CI = 1.17–1.44). In contrast, alcohol consumption (OR = 0.88, 95%CI = 0.56–1.36), smoking (OR = 0.77, 95%CI = 0.47–1.27), a relative with CCA (OR = 1.07, 95%CI = 0.36–1.20), defecation in the latrine (OR = 1, 95%CI = 0.44–0.56), agriculture and pesticide use (OR = 5.12, 95%CI = 0.59–44.40) and a house near wetlands (OR = 0.97, 95%CI = 0.61–1.56) were not factors associated with *O. viverrini* infection.

## 4. Discussion

This report provided the first evidence that drew attention to the prevalence of *O. viverrini* infection cases during the four years of study in this region. The study area in Sakon Nakhon province, northeastern Thailand has high morbidity and mortality due to *O. viverrini* infection and CCA [8]. The current results showed that the prevalence rate and risk factors of *O. viverrini* increased during 2017–2020. The overall prevalence rates of *O. viverrini* infection in the subjects were 2.47%, 3.60%, 5.21%, and 7.01% during 2017, 2018, 2019, and 2020, respectively. Recently, Srithai et al., [21] reported that the prevalence of *O. viverrini* in the Phon Sawan District of Nakhon Phanom Province, Thailand was 24%, and almost half of respondents (49.2%) also reported consuming raw fish in this region. In addition, the parasitic persistency of *O. viverrini* infection has also been reported in animal reservoir hosts, such as cats and dogs [25] and intermediated hosts [22]. The prevalence of *O. viverrini* infection in the study was predominantly in women, which was different from other studies in northeastern Thailand [10,21,26,27]. These other results were suggested to be due to men consuming more *koi-pla* or *lab-pla* with alcoholic beverages [9,26]. However, in the current study, women were also at risk of *O. viverrini* infection, perhaps because the factors of alcohol consumption and smoking were not found to be associate factors, whereas status was a significant risk factor in the current study. Because of residents among rural households always sharing food with community members, this can increase the frequency of raw fish consumption and the risk of acquiring a liver fluke infection [28]. Eating raw freshwater fish by adding lime juice or drinking alcohol is erroneously thought to kill the parasites in the dish [29]. Previous studies reported that alcohol consumption was associated with an increased chance of acquiring *O. viverrini* infection [30].

This study revealed that females are also at a significant risk for *O. viverrini* infection. The history of praziquantel administration was a significant risk factor of *O. viverrini* infection in this study, perhaps because the belief in its effectiveness encouraged people to continue eating the traditional northeastern raw fish dishes [31]. The treatment of praziquantel was associated with increasing *O. viverrini* infection. The reinfection rate of liver fluke infection was 10.9% in 457 subjects [31]. Thinkhamrop et al. [32] conducted a cross-sectional study in northeastern Thailand and reported that repeated praziquantel administration was significantly associated with increasing *O. viverrini* infection. The odds ratio of participants who used the drug once was 1.09, it was 1.19 for those using it twice, it was 1.28 for those using it three times, and it was 1.86 for those using it more than three times when compared with participants who never used praziquantel [32]. This is similar to previous findings among *O. viverrini* infection have been observed to history of praziquantel treatment [32,33,34,35]. However, people are still continuing to consume raw or uncooked freshwater fish because they know that praziquantel is available to kill the parasite resulting to reinfection of *O. viverrini* and its potential long-term risks [36].

The major problem for *O. viverrini* infection control in northeastern Thailand is the unhygienic behavior and lack of education about the risks of consuming traditionally prepared fish dishes by the rural population [37]. In this region, ethnic Issan-Lao comprise 80% of the population, having a distinct language, ancestry, and cultural food preferences [37]. Opisthorchiasis is a neglected disease affecting the low-income people in this region according to Sripa [38], who also reported similar family incomes of about 5001–10,000 Thai Baht per month. People in this region are likely to eat various uncooked items, such as raw fish and fermented fish, and semi-cooked items, such as koi-pla, lab-pla, som-khai pla, pla-som, and pla-ra, as traditional staple foods together with sticky rice [27,37]. The type of raw fish in the *O. viverrini* infection cases in this study was commonly koi-pla and lab-pla. The reason for eating raw food was due to habit rather than being addicted to the taste. Among these habits, consumption of raw or undercooked fish was demonstrated to be a significant contributing factor to the prevalence of *O. viverrini* in the present study; similar other published studies [29,33,39,40,41]. 

In 2009, *O. viverrini* was classified as a group one biological carcinogen capable of causing cancer in humans by the International Agency for Research on Cancer (IARC) and the World Health Organization (WHO) [10]. Additional risk factors may be associated with CCA via the disruption of anti-inflammatory mechanisms or as inflammatory agents [37]. Poverty has been a significant risk factor resulting in chronic disease through chronic psychosocial stress leading to alcohol consumption and other risk behaviors [37,42]. In northeastern Thailand, rural poverty has persisted despite government efforts [43]. Toxic chemical exposure is a major risk factor for liver cancer, including from pesticides and herbicides [35]. Farmers use the herbicide glyphosate for rice and particularly sugar cane production, and they have limited knowledge about its human health risks and effects on the environment and associated mitigation strategies [44]. However, agriculture and pesticide use (OR = 5.12, 95%CI = 0.59–44.40) were identified as a risk factor for this infection, although it was not significant (*p*-value 0.138). The current study had some limitations. The questionnaire regarding probable risk factors for *O. viverrini* infections did not cover sociodemographic information such as knowledge and attitude.

## 5. Conclusions

In conclusion, our findings have identified the risk factor of *O. viverrini* infection in Sakon Nakhon province, Thailand. Factors associated with *O. viverrini* infection were gender and status. In addition, the health behavior factors were the habit of consuming unsafely prepared fish, the medical history of *O. viverrini* examination, a history of *O. viverrini* infection, and a history of taking praziquantel. Although the risk factors for *O. viverrini* infection and CCA development in northeastern Thailand have been well-documented, relevant incidences show no declining trend over recent decades. Improved knowledge to stop people from eating raw or undercooked fish or to encourage studying food safety procedures, diminishing the health risk from agrochemical toxicity through health promotion, or waiting for a new generation of people in this region are considered to be key factors for effective and sustainable control of *O. viverrini* infection and CCA in northeastern Thailand.

## Figures and Tables

**Table 1:** Baseline characteristics of 320 participants.

Variable	Controls (n = 160)	Cases (n = 160)	Total	Univariate OR	95%CI	*p*-Value
	Number (%)	Number (%)	320			
Gender						
Male	84 (52.50)	66 (41.25)	150 (46.90)	1		
Female	76 (47.50)	94 (58.75)	170 (53.10)	1.23 *	0.84–1.15	0.044
Age (years)						
55+	108 (67.50)	99 (61.87)	207 (64.70)	1		
≤55	52 (32.50)	61 (38.13)	113 (35.30)	1.50	0.86–1.18	0.294
Status						
Widowed/						
divorced/						
separated	18 (11.25)	7 (4.37)	25 (7.80)	1		
Married	142 (88.75)	153 (95.62)	295 (92.20)	8.27 *	0.55–1.05	0.022
Education						
Secondary school/upper						
	16 (10.00)	13 (8.10)	29 (9.10)	1		
Primary school/lower						
	144 (90.00)	147 (91.90)	291 (90.90)	1.28	0.71–1.19	0.560
Occupation						
Other	17 (10.60)	9 (5.60)	26 (8.10)	1		
Agriculture	143 (89.40)	151 (94.40)	294 (91.90)	2.11	0.61–1.11	0.102
Family income per month (THB)						
<5000	39 (24.40)	28 (17.50)	67 (20.90)	1		
5001–10,000	91 (56.90)	97 (60.60)	188 (58.80)	1.16	1.33–2.50	0.277
10,001–15,000	21 (13.10)	29 (18.10)	50 (15.60)			
>15,000	9 (5.60)	6 (3.80)	15 (4.70)			

* *p*-value < 0.05.

**Table 2:** Behavior factors associated with *O. viverrini* infection in Sakon Nakhon province, northeastern Thailand.

Variable	Controls (n = 160)	Cases (n = 160)	Total	Univariate OR	95%CI	*p*-Value
	Number (%)	Number (%)	320			
Habit of eating raw fish						
Several times	30 (18.75)	95 (59.38)	125 (39.06)	1		
Sometimes	130 (81.25)	65 (40.62)	195 (60.94)	6.33 ***	0.32–0.59	<0.0001
History of OV examination						
Never	138 (86.25)	66 (41.25)	204 (63.75)	1		
1st time	22 (13.75)	94 (58.75)	116 (36.25)	8.93 ***	5.15–15.47	<0.0001
History of OV infection						
Never	160 (100.00)	71 (44.38)	231 (72.20)	1		
Ever	0 (0)	89 (55.62)	89 (27.80)	3.64 ***	1.17–1.44	<0.0001
History of praziquantel administration						
Never use	160 (100.00)	71 (44.37)	231 (72.20)	1		
Have used	0 (0)	89 (55.63)	89 (27.80)	3.64 ***	1.17–1.44	<0.0001
Relative with CCA						
None	129 (80.62)	138 (86.25)	267 (83.43)	1		
Have relative	31 (19.38)	22 (13.75)	53 (16.57)	1.07	0.36–1.20	0.175
Alcohol consumption						
No	84 (52.50)	89 (55.62)	173 (54.06)	1		
Yes	76 (47.50)	71 (44.38)	147 (45.94)	0.88	0.56–1.36	0.575
Smoking						
No	113 (70.63)	121 (75.63)	234 (71.13)	1		
Yes	47 (29.37)	39 (24.37)	86 (26.87)	0.77	0.47–1.27	0.314
Defecation in latrine						
No	0 (0)	0 (0)	0 (0)	1		
Yes	160 (100.00)	160 (100.00)	320 (100.00)	1.00	0.44–0.56	1.00
Agriculture and pesticide used						
Never used	159 (99.38)	155 (96.88)	314 (98.13)	1		
Have used	1 (0.62)	5 (3.12)	6 (1.87)	5.12	0.59–44.40	0.138
House near wetlands						
No	110 (68.75)	111 (69.37)	221 (69.07)	1		
Yes	50 (31.25)	49 (30.63)	99 (30.93)	0.97	0.61–1.56	0.904

*** *p* < 0.0001.

## Data Availability

The datasets used and/or analyzed during the current study are available from the corresponding author on reasonable request.
