# Correction: Perakanya et al. Prevalence and Risk Factors of *Opisthorchis viverrini* Infection in Sakon Nakhon Province, Thailand. *Trop. Med. Infect. Dis.* 2022, *7*, 313

**DOI:** 10.3390/tropicalmed8040222

**Published:** 2023-04-11

**Authors:** Pariyakorn Perakanya, Ratchadaporn Ungcharoen, Sutthiporn Worrabannakorn, Passakorn Ongarj, Atchara Artchayasawat, Thidarut Boonmars, Parichart Boueroy

**Affiliations:** 1 Department of Community Health, Faculty of Public Health, Chalermphrakiat Sakon Nakhon Campus, Kasetsart University, Sakon Nakhon 47000, Thailand; 2 Cholangiocarcinoma Research Center, Sakon Nakhon Hospital, Sakon Nakhon 47000, Thailand; 3 Department of Parasitology, Faculty of Medicine, Khon Kaen University, Khon Kaen 40002, Thailand

The authors wish to make the following corrections to this paper [1], in the context of OR and 95%CI as presented in Abstract, Table 1 and Table 2 and Results. These mistakes have been resolved. The authors state that the scientific conclusions are unaffected. This correction was approved by the Academic Editor. The original publication has also been updated. The authors would like to apologize for any inconvenience caused to the readers by these changes.

Corrected the OR and 95%CI in Abstract as follow, replace:

“Factors associated with *O. viverrini* infection were the habit of consuming unsafely prepared fish (OR = 6.33, 95%CI = 0.32–0.59), the medical history of *O. viverrini* examination (OR = 8.93, 95%CI = 5.15–15.47), a history of *O. viverrini* infection (OR = 3.64, 95%CI = 1.17–1.44), and a history of taking praziquantel (OR = 3.64, 95%CI = 1.17–1.44)”.

with

“Factors associated with *O. viverrini* infection were the habit of consuming unsafely prepared fish (OR = 6.33, 95%CI = 3.71–10.90), the medical history of *O. viverrini* examination (OR = 8.93, 95%CI = 5.15–16.21), a history of *O. viverrini* infection (OR = 201.25, 95%CI = 33.32–8082.76), and a history of taking praziquantel (OR = 201.25, 95%CI = 33.32–8082.76)”.

Corrected the OR and 95%CI in Table 1 as follow.

**Table 1:** Baseline characteristics of 320 participants.

Variable	Controls (n = 160)	Cases (n = 160)	Total	Univariate OR	95%CI	*p*-Value
	Number (%)	Number (%)	320			
Gender						
Male	84 (52.50)	66 (41.25)	150 (46.90)	1		
Female	76 (47.50)	94 (58.75)	170 (53.10)	1.57 *	0.99–2.51	0.044
Age (years)						
55+	108 (67.50)	99 (61.87)	207 (64.70)	1		
≤55	52 (32.50)	61 (38.13)	113 (35.30)	1.28	0.79–2.08	0.294
Status						
Widowed/						
divorced/						
separated	18 (11.25)	7 (4.37)	25 (7.80)	1		
Married	142 (88.75)	153 (95.62)	295 (92.20)	2.77 *	1.06–8.06	0.022
Education						
Secondary school/upper						
	16 (10.00)	13 (8.10)	29 (9.10)	1		
Primary school/lower						
	144 (90.00)	147 (91.90)	291 (90.90)	1.26	0.55–2.95	0.560
Occupation						
Other	17 (10.60)	9 (5.60)	26 (8.10)	1		
Agriculture	143 (89.40)	151 (94.40)	294 (91.90)	2.00	0.81–5.24	0.102
Family income per month (THB)						
<5000	39 (24.40)	28 (17.50)	67 (20.90)	1		
5001–10,000	91 (56.90)	97 (60.60)	188 (58.80)	0.67	0.37–1.23	0.17
10,001–15,000	21 (13.10)	29 (18.10)	50 (15.60)	0.52	0.23–1.16	0.08
>15,000	9 (5.60)	6 (3.80)	15 (4.70)	1.08	0.30–4.12	0.89

* *p*-value < 0.05

Corrected the OR and 95%CI in Table 2 as follow.

**Table 2:** Behavior factors associated with O. viverrini infection in Sakon Nakhon province, northeastern Thailand.

Variable	Controls (n = 160)	Cases (n = 160)	Total	Univariate OR	95%CI	*p*-Value
	Number (%)	Number (%)	320			
Habit of eating raw fish						
Several times	30 (18.75)	95 (59.38)	125 (39.06)	1		
Sometimes	130 (81.25)	65 (40.62)	195 (60.94)	6.33 ***	3.71–10.90	<0.0001
History of OV examination						
Never	138 (86.25)	66 (41.25)	204 (63.75)	1		
1st time	22 (13.75)	94 (58.75)	116 (36.25)	8.93 ***	5.15–16.21	<0.0001
History of OV infection						
Never	160 (100.00)	71 (44.38)	231 (72.20)	1		
Ever	0 (0)	89 (55.62)	89 (27.80)	201.25 ***	33.32–8082.76	<0.0001
History of praziquantel administration						
Never use	160 (100.00)	71 (44.37)	231 (72.20)	1		
Have used	0 (0)	89 (55.63)	89 (27.80)	201.25 ***	33.32–8082.76	<0.0001
Relative with CCA						
None	129 (80.62)	138 (86.25)	267 (83.43)	1		
Have relative	31 (19.38)	22 (13.75)	53 (16.57)	1.07	0.36–1.20	0.175
Alcohol consumption						
No	84 (52.50)	89 (55.62)	173 (54.06)	1		
Yes	76 (47.50)	71 (44.38)	147 (45.94)	0.88	0.56–1.36	0.575
Smoking						
No	113 (70.63)	121 (75.63)	234 (71.13)	1		
Yes	47 (29.37)	39 (24.37)	86 (26.87)	0.77	0.47–1.27	0.314
Defecation in latrine						
No	0 (0)	0 (0)	0 (0)	1		
Yes	160 (100.00)	160 (100.00)	320 (100.00)	-	-	-
Agriculture and pesticide used						
Never used	159 (99.38)	155 (96.88)	314 (98.13)	1		
Have used	1 (0.62)	5 (3.12)	6 (1.87)	5.12	0.59–44.40	0.138
House near wetlands						
No	110 (68.75)	111 (69.37)	221 (69.07)	1		
Yes	50 (31.25)	49 (30.63)	99 (30.93)	0.97	0.61–1.56	0.904

*** *p* < 0.0001.

Corrected the OR and 95%CI in Result as follow, replace:

“Table 1 shows the univariate analysis results for the general factors contributing to the risk of infection with *O. viverrine* Age (OR = 1.50, 95%CI = 0.86–1.18), education level (OR = 1.28, 95%CI = 0.71–1.19), occupation (OR = 2.11, 95%CI = 0.61–1.11), and family income (OR = 1.16, 95%CI = 1.33–2.50) were not found to be risk factors, while gender (OR = 1.23, 95%CI = 0.84–1.15) and status (OR = 8.27, 95%CI = 0.55–1.05) were significant factors regarding *O. viverrini* infection in Sakon Nakhon province, northeastern Thailand”.

with

“Table 1 shows the univariate analysis results for the general factors contributing to the risk of infection with *O. viverrini* Age (OR = 1.28, 95%CI = 0.79–2.08), education level (OR = 1.26, 95%CI = 0.55–2.95), occupation (OR = 2.00, 95%CI = 0.81–5.2), and family income was THB 5001–10,000/month (OR = 0.67, 95%CI = 0.37–1.23), THB 10,001–15,000/month (OR = 0.52, 95%CI = 0.23–1.16), and THB > 15,000/month (OR = 1.08, 95%CI = 0.30–4.12) were not found to be risk factors, while gender (OR = 1.57, 95%CI = 0.99–2.51) and status (OR = 2.77, 95%CI = 1.060–8.06) were significant factors regarding *O. viverrini* infection in Sakon Nakhon province, northeastern Thailand”.

Corrected the OR and 95%CI in Results as follow, replace:

“The associated factors of *O. viverrini* infection in Sakon Nakhon province, northeastern Thailand were the habit of eating raw fish (OR = 6.33, 95%CI = 0.32–0.59), a history of *O. viverrini* examination (OR = 8.93, 95%CI = 5.15–15.47), a history of *O. viverrini* infection (OR = 3.64, 95%CI = 1.17–1.44), and a history of taking praziquantel (OR = 3.64, (95%CI = 1.17–1.44). In contrast, alcohol consumption (OR = 0.88, 95%CI = 0.56–1.36), smoking (OR = 0.77, 95%CI = 0.47–1.27), a relative with CCA (OR = 1.07, 95%CI = 0.36–1.20), defecation in the latrine (OR = 1, 95%CI = 0.44–0.56), agriculture and pesticide use (OR = 5.12, 95%CI = 0.59–44.40) and a house near wetlands (OR = 0.97, 95%CI = 0.61–1.56) were not factors associated with *O. viverrini* infection”.

with

“The associated factors of *O. viverrini* infection in Sakon Nakhon province, northeastern Thailand were the habit of eating raw fish (OR = 6.33, 95%CI = 3.71–10.90), a history of *O. viverrini* examination (OR = 8.93, 95%CI = 5.15–16.215), a history of *O. viverrini* infection (OR = 201.25, 95%CI = 33.32–8082.76), and a history of taking praziquantel (OR = 201.25, (95%CI = 33.32–8082.76). In contrast, alcohol consumption (OR = 0.88, 95%CI = 0.56–1.36), smoking (OR = 0.77, 95%CI = 0.47–1.27), a relative with CCA (OR = 1.07, 95%CI = 0.36–1.20), defecation in the latrine, agriculture and pesticide use (OR = 5.12, 95%CI = 0.59–44.40) and a house near wetlands (OR = 0.97, 95%CI = 0.61–1.56) were not factors associated with *O. viverrini* infection”.
